# Characterization of the imprinting signature of mouse embryo fibroblasts by RNA deep sequencing

**DOI:** 10.1093/nar/gkt1042

**Published:** 2013-11-11

**Authors:** Diana A. Tran, Angela Y. Bai, Purnima Singh, Xiwei Wu, Piroska E. Szabó

**Affiliations:** ^1^Department of Molecular and Cellular Biology, Beckman Research Institute, City of Hope National Medical Center, Duarte, CA 91010, USA, ^2^Irell and Manella Graduate School of Biological Sciences, Beckman Research Institute, City of Hope National Medical Center, Duarte, CA 91010, USA, ^3^Eugene and Ruth Roberts Summer Academy, City of Hope National Medical Center, Duarte, CA 91010, USA and ^4^Department of Molecular Medicine, Beckman Research Institute, City of Hope National Medical Center, Duarte, CA 91010, USA

## Abstract

Mouse embryo fibroblasts (MEFs) are convenient sources for biochemical studies when cell number in mouse embryos is limiting. To derive the imprinting signature of MEFs and potentially detect novel imprinted genes we performed strand- and allele-specific RNA deep sequencing. We used sequenom allelotyping in embryo and adult organs to verify parental allele-specific expression. Thirty-two known ubiquitously imprinted genes displayed correct parental allele-specific transcripts in MEFs. Our analysis did not reveal any novel imprinted genes, but detected extended parental allele-specific transcripts in several known imprinted domains: maternal allele-specific transcripts downstream of *Grb10* and downstream of *Meg3*, *Rtl1as* and *Rian* in the *Dlk1-Dio3* cluster, an imprinted domain implicated in development and pluripotency. We detected paternal allele-specific transcripts downstream of *Nespas*, *Peg3, Peg12* and *Snurf/Snrpn*. These imprinted transcript extensions were not unique to MEFs, but were also present in other somatic cells. The 5′ end points of the imprinted transcript extensions did not carry opposing chromatin marks or parental allele-specific DNA methylation, suggesting that their parental allele-specific transcription is under the control of the extended imprinted genes. Based on the imprinting signature of MEFs, these cells provide valid models for understanding the biochemical aspects of genomic imprinting.

## INTRODUCTION

Imprinted genes exhibit allele-specific transcription depending on parental origin of the chromosomes (1). Imprinted genes play important roles in development, and their monoallelic expression poses increased risk upon their damage or loss (2). Apart from protein-coding genes, imprinted long non-coding RNAs have important functions in regulating domain-wide imprinted expression (3–5) and imprinted small RNAs can modulate the transcription of non-imprinted genes (6–9). Parental allele-dependent expression of imprinted genes is ubiquitous in most cases but can also be tissue-specific and may depend on developmental stage (10). The number of imprinted genes in different mammalian species is around 100 (11). Over 1300 imprinted genes were reported in the mouse brain (12), but the validity of this finding is debated (13–15). Mouse embryo fibroblasts (MEFs) are convenient sources for biochemical studies. To assess the validity of this model system for studying genomic imprinting and potentially detect novel imprinted genes we derived the imprinting signature of MEFs, generated from reciprocal mouse crosses, using strand- and allele-specific RNA deep sequencing. We confirmed parental allele-specific expression of 32 known ubiquitously imprinted genes, detected extended allele-specific transcripts downstream of 8 known imprinted genes, but did not find any novel imprinted transcripts in MEFs.

## MATERIALS AND METHODS

### Generation of MEFs, mouse embryos and adult organs

JF1XOG2 (JXO) embryos were generated using the inbred JF1/Ms inbred strain (purchased from The Jackson Laboratory) and the TgOG2 (16) transgenic line (that has been maintained in our laboratory as a small stock since 2002) as mother and father, respectively. OG2XJF1 (OXJ) embryos were generated using the reciprocal mating. MEFs were derived from 13.5 dpc embryos using standard procedures. Briefly, the head and internal organs were removed from the embryo, and the remaining carcass was dispersed to single cell suspension by Trypsin digestion and trituration. The sex of the embryo was determined by the morphology of the gonad, which is clearly distinct at this stage. The cell suspension resulting from each embryo was plated on a 15 cm tissue culture dish and, after reaching confluence it was cryopreserved in five vials per embryo. Passage 2 or 3 was used for the RNA analysis. RNA was also prepared for allelotyping from organs of adult (8 weeks old) male and female mice resulting from JF1X129 and reciprocal 129XJF1 crosses from 129S1 and JF1 parental strains.

### RNA isolation

RNA was isolated using RNA-Bee (Tel-Test Inc.). DNAse I digestion was applied to remove DNA contamination. Ten microgram of total RNA was treated with the Ribominus kit (Life Technologies) to remove ribosomal RNA contamination.

### Deep sequencing

Sample preparation and deep sequencing was done from one JXO and one OXJ MEF line according to Illumina protocols with proper clean up between each step. Briefly, first-strand synthesis was done using Superscript II reverse transcriptase, dNTPs and the Illumina RNA seq library preparation kit. The enzyme was inactivated, and the dNTPs were removed. To enable strand-specific analysis, the second strand cDNA was labeled with dUTP before ligating the adapter. The second strand was synthesized using *Escherichia coli* DNA polymerase I and dATP, dGTP, dCTP, dUTP nucleotides in the presence of *E. coli* ligase and RNase H. Ends were repaired using ‘End It Enzyme mix’, and dATP 3′ overhangs were created using Klenow exo- and then R2 adapters were ligated to the double-stranded cDNA fragments. The second strand was degraded using USER™ (Uracil-Specific Excision Reagent) enzyme complex. Only the first strand was used as PCR template for paired-end deep sequencing. The read 1 sequences (F2R1 or R1F2) represent the antisense strand, and the read 2 (F1R2 or R2F1) sequences represent the sense strand of the RNA. The single-stranded cDNA containing the adapter was amplified by 15 cycles of PCR using two different index primers. Reads resulting from the JXO cross MEF started with the sequence ACTTGA (AC) and reads resulting from the OXJ cross MEF started with the sequence CCGTCC (CC). The two MEF samples were sequenced on half a lane each of the Illumina sequencer.

### Bioinformatics

Sequencing reads were aligned to the mm9 genome with Tophat v1.2.0 using default parameters with ‘-g 10’ to allow the reads to align to multiple loci. This is stricter than the default setting but still ensures that alternative exons are not excluded. Regions that are highly repetitive (more than 10 copies in the genome) will not be detected by this option. Aligned reads were used to generate pileup for each base with Samtools v0.1.18. The pileup reads were used to identify the single nucleotide polymorphisms (SNPs) that are present in either AC or CC with ≥80% alternative allele frequency (AF) and total coverage ≥10. Only bases with quality score ≥13 were considered. The reference allele and alternative alleles in either AC or CC samples were then counted for these candidate SNPs, and SNPs with ≥80% in both AC and CC were excluded. The *P*-value and odds ratio were calculated using Fisher’s exact test based on the coverage information of reference and alternative alleles in AC and CC samples, and *P*-value was adjusted by false discovery rate (FDR) method (17). Final SNPs were selected if FDR ≤0.05, and the reciprocal sample has alternative AF ≤20%. The SNPs were annotated with mm9 refseq database downloaded 05/05/12.

### Verification of imprinted expression by sequenom allelotyping

We designed multiplex sequenom allelotyping assays using the SNPs derived from the deep sequencing analysis, including known and putative imprinted genes. Sequenom allelotyping was performed as we described earlier (18) using cDNA from embryo and adult organs and MEFs. The primer sequences are listed in Supplementary Table S1.

### RACE analysis

We used SMARTer™ RACE cDNA amplification kit (Clontech) and primers 5′-TGGGTGGATCGTACCTCGGCCTAA-3′ and 5′-CCTGTGAAAAGCAAACTGAGGCGAGA-3′ to find the 3′ end of *Rian* and potential 5′ end of *Rian* extension, respectively. The 3′ end of *Rian* was correctly amplified, but no PCR product was obtained from the 5′ end of *Rian* extension. A 3-kb long PCR product was obtained between *Rian* and *Rian* extension, which was subcloned and verified by DNA sequencing.

### Methylated CpG island recovery assay and MIRA-SNuPE

The methylated fraction of sonicated genomic DNA from MEFs was captured using recombinant MBD2b and MBD3L1 proteins as described earlier (19). Parental allele-specific CpG methylation was measured using multiplex single nucleotide extension (SNuPE) by sequenom allelotyping assays (20). The primer sequences are listed in Supplementary Table S1.

### Chromatin immunoprecipitation and ChIP-chip

For chromatin immunoprecipitation, female and male GFP-negative somatic cells were used. These were collected by FACS from CF1XOG2 embryo gonads as described previously (21) based on *Pou5f1* promoter-driven EGFP expression in germ cells but not in somatic cells (16) using a MoFlo or Aria III flow cytometer. ChIP was performed as described previously (22,23). Chromatin from 400 000 cells was used for one ChIP. Custom-designed tiling arrays (110228_MM9_PS_ChIP), manufactured by Roche/NimbleGen, were used for the histone modification profile analysis. Amplified ChIP DNA fractions were compared with amplified input DNA. Data were extracted from scanned images by using NimbleScan 2.3 extraction software (NimbleGen Systems). Primary ChIP-chip data (22) had been deposited to GEO database with SuperSeries accession number GSE46954.

## RESULTS

### Deep sequencing of MEF RNA from reciprocal mouse crosses

To characterize the imprinting signature of MEFs, we performed allele-specific and strand-specific paired-end RNA deep sequencing using Illumina deep sequencer. To achieve high coverage of SNPs between the parental alleles we mated female inbred JF1/Ms (JF1) mice with male TgOG2 (OG2) transgenic mice (JXO cross), and by switching the parents we generated the reciprocal (OXJ) cross. The OG2 transgenic line was originally made by injecting (CBA/CaJ × C57BL/6J)F_2_ zygotes and subsequent breeding to homozygosity for the GOF18ΔPE-EGFP transgene (16). Both the CBA/CaJ and the C57BL/6J inbred strains belong to the *Mus musculus* domesticus subspecies. The genetically distinct JF1 inbred strain belongs to the *M. musculus* molossinus subspecies (24) and provides over 15 million SNPs compared to the C57BL/6J reference genome (25). We prepared RNA at early passage numbers (passage 2–3) from female 13.5 dpc JXO and OXJ embryos for deep sequencing.

Sequencing reads were aligned to the mm9 genome with Tophat v1.2.0. We obtained 95 517 702 and 117 618 284 total reads resulting 76 712 608 (80.3%) and 86 369 632 (73.4%) aligned reads from the JXO and OXJ MEFs, respectively. In total, 90.2% and 89.1% of the aligned reads were in the sense orientation along exons (coding and non-coding combined). The aligned reads were used to generate pileup for each base with Samtools v0.1.18. The pileup reads were used to identify the SNPs that are present in either JXO or OXJ with ≥80% alternative AF and total coverage ≥10. The reference allele and alternative alleles in either JXO or OXJ samples were then counted for these candidate SNPs. SNPs with ≥80% in both JXO and OXJ were excluded. The *P*-value and odds ratio were calculated using Fisher’s exact test based on the coverage information of reference and alternative alleles in the JXO and OXJ samples, and *P*-values were adjusted by FDR method (17). A total of 1247 predictive (TRUE) SNPs (Supplementary Table S2) were selected based on the following cutoff criteria: the FDR in one sample was <0.05 and the reciprocal sample had an alternative AF <20%. Out of the 1247 TRUE SNPs, 813 and 434 SNPs qualified in the JXO and OXJ MEFs, respectively. The number of SNPs below this cutoff (FALSE) was 17 858 total (11 800 in JXO and 6058 in OXJ MEFs).

There is a substantial discrepancy between the numbers of TRUE SNPs in the two crosses. This is due to our method of identifying SNPs. The JXO and OXJ datasets were complementary: one cross was used for calling each TRUE SNP (alternative JF1 allele is in abundance), and the other cross was used to confirm it (alternative JF1 allele is in minority). As one can see from Supplementary Table S2, the JXO cross has revealed maternally expressed imprinted genes and the OXJ cross confirmed them. On the other hand, the OXJ cross has revealed paternally expressed imprinted genes and the JXO cross confirmed them. Therefore, the number of TRUE SNPs in the JXO or OXJ cross, respectively, only depends on the number of maternally or paternally expressed imprinted transcripts, respectively, and the number of SNPs along their lengths.

### Confirming known imprinted genes

The SNPs were annotated with mm9 refseq database. Of 1251 TRUE SNPs, 861 mapped to 32 known imprinted genes (Table 1), many of these are known ubiquitously imprinted. Transcription was found in the correct DNA strand (Table 1; Supplementary Table S2). For example *Igf2* and *Igf2as* transcripts occurred from (−) and (+) DNA strands, respectively. Imprinted transcripts usually harbored more than one of the allele-specific SNPs. *Grb10* and *Kcnq1ot1* had the most support with 205 and 201 SNPs, respectively. The OG2 strain has performed well against the JF1 inbred strain in identifying imprinted transcripts. The OG2 sequence aligned in general with the C57BL/6J reference genome. For the known maternally expressed ubiquitous imprinted genes, 502 of 527 (95%) SNPs identified the JF1 as alternative allele (in the JXO cross). For the paternally expressed ubiquitous imprinted genes 330 of 334 SNPs (99%) identified JF1 as alternative allele (in the OXJ cross), and each of these calls were confirmed in the reciprocal cross (Table 1; Supplementary Table S2).

**Table 1.** Known imprinted genes confirmed in MEF deep sequencing

Transcript	Number of SNPs	Average reads	Strand	Representative SNP	JXO alt SNP	JXO ref SNP	OXJ ref SNP	OXJ alt SNP	AF JXO	AF OXJ	FDR	Sequenom results	Notes on IGV
*AF357425*	13	32	+	chr12_110872195	47	0	47	0	1.00	0.00	1.52E-23		MAT
*AK050713*	12	27	+	chr12_110907826	31	0	22	0	1.00	0.00	2.64E-11		MAT
*Asb4*	7	19	+	chr6_5348300	19	0	9	2	1.00	0.18	0.045042863	MAT	MAT
*Cdkn1c*	3	129	−	chr7_150644367	202	1	133	0	1.00	0.00	4.16E-91	MAT	MAT
*Grb10*	205	106	−	chr11_11831012	1789	7	1600	4	1.00	0.00	0	MAT	MAT
*H19*	6	7323	−	chr7_149761651	7298	4	5969	8	1.00	0.00	0	MAT	MAT
*Igf2r*	14	136	−	chr17_12876894	167	3	244	5	0.98	0.02	1.22E-102	MAT	MAT
*Meg3*	20	133	+	chr12_110783337	139	0	118	0	1.00	0.00	2.55E-72	MAT	MAT
*Mirg*	66	35	+	chr12_110980023	56	0	39	0	1.00	0.00	1.74E-23		MAT
*Rian*	202	98	+	chr12_110884294	926	10	712	0	0.99	0.00	0	MAT	MAT
*Rtl1as*	2	18	+	chr12_110831619	20	0	22	0	1.00	0.00	2.36E-08	MAT	MAT
*Airn*	1	13	+	chr17_13008045	0	13	0	12	0.00	1.00	0.001142434	PAT	PAT
*Blcap*	1	55	−	chr2_157387807	3	42	0	65	0.00	0.93	1.98E-23		PAT
*D7Ertd715e*	5	14	−	chr7_67115005	0	13	0	17	0.00	1.00	5.01E-05	PAT	PAT
*Igf2*	6	1706	−	chr7_149836880	18	2774	0	2589	0.00	0.99	0	PAT	PAT
*Igf2as*	2	31	+	chr7_149853327	0	36	0	55	0.00	1.00	2.22E-22	PAT	PAT
*Impact*	14	187	+	chr18_13133281	23	362	10	292	0.03	0.94	3.05E-146	PAT	PAT
*Kcnq1ot1*	201	22	−	chr7_150427528	0	14	0	22	0.00	1.00	1.60E-06	PAT	PAT
*Mest*	1	380	+	chr6_30695854	9	300	0	450	0.00	0.97	8.79E-200		PAT
*Ndn*	1	67	+	chr7_69493343	0	59	0	74	0.00	1.00	1.97E-35		PAT
*Nespas*	2	11	−	chr2_174107316	0	12	0	9	0.00	1.00	0.020011567	PAT	PAT
*Nnat*	4	79	+	chr2_157387776	2	48	1	77	0.01	0.96	8.59E-28	PAT	PAT
*Peg10*	10	115	+	chr6_4707869	0	115	0	94	0.00	1.00	4.06E-58	PAT	PAT
*Peg12*	3	39	−	chr7_69608449	0	44	0	47	0.00	1.00	3.23E-23	PAT	PAT
*Peg13*	5	31	−	chr15_72639823	0	15	0	52	0.00	1.00	1.80E-11	PAT	PAT
*Peg3*	17	53	−	chr7_6662661	1	49	0	42	0.00	0.98	9.36E-22	PAT	PAT
*Plagl1*	18	45	+	chr10_12844715	0	46	0	47	0.00	1.00	7.81E-24	PAT	PAT
*Sgce*	4	81	−	chr6_4639630	0	103	0	108	0.00	1.00	3.78E-59		PAT
*Slc38a4*	19	350	−	chr15_96825404	2	143	41	355	0.10	0.99	4.89E-86	PAT	PAT
*Snrpn*	1	45	−	chr7_67133548	2	31	0	56	0.00	0.94	3.91E-18	PAT	PAT
*Snurf*	1	45	−	chr7_67133548	2	31	0	56	0.00	0.94	3.91E-18	PAT	PAT
*Zdbf2*	17	24	+	chr1_63360418	0	11	0	35	0.00	1.00	4.56E-07	PAT	PAT

We tabulated in alphabetical order the previously known imprinted transcripts that were confirmed in our RNA deep sequencing experiments using MEFs. We provided the number of informative SNPs and the average read number per SNP along each transcript. The direction of the transcript is marked with regard to the two (+ and −) DNA strands, as it appears in the UCSC browser. We also included a representative SNP from each imprinted transcript with chromosomal coordinate, and read numbers at this SNP for the reference (OG2) and the alternative (JF1) allele in the JF1xOG2 and OG2xJF1 (JXO and OXJ) crosses, where the mother’s genotype is always written first. The calculated AF follows for the alternative allele (maternal and paternal allele, respectively) in the reciprocal crosses. The FDR of the predictor algorithm was calculated based on the AF values and statistical significance (see ‘Materials and Methods’). The results of sequenom allelotyping experiments (when tested) and visual inspection of the transcripts using IGV are indicated by the transcribed parental allele maternal (MAT) or paternal (PAT).

**Figure 1.**
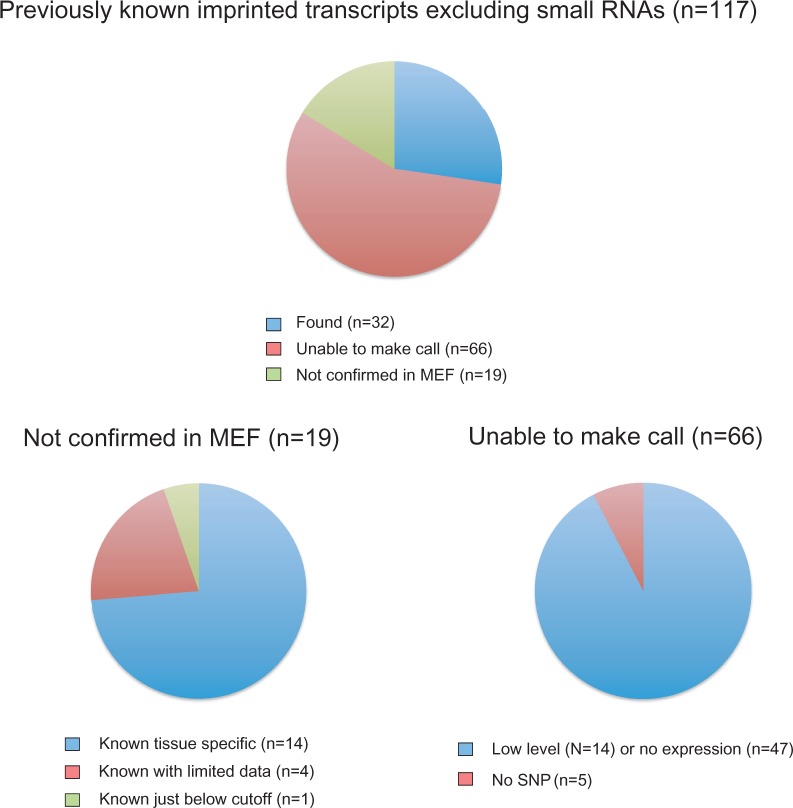
Detecting known imprinted genes by RNA deep sequencing in MEFs. Known imprinted genes are tabulated into groups of found, not confirmed in MEF and unable to make call. The latter categories are divided further. See details in Supplementary Table S3.

We summarized the reasons for not detecting other known imprinted genes (Figure 1; Supplementary Table S3). Of 152 known imprinted transcripts (26) we excluded 22 small RNAs, because our method is not suited for small RNA detection and also excluded 12 coding transcripts that were recently shown not to be genuinely imprinted (15), and one that was a duplicate entry (A19). Of the remaining 117 known imprinted genes we were unable to make a call for 66 genes, either because of the low level or no transcription in MEF (61 transcripts), or lack of SNPs (five transcripts). Nineteen known imprinted genes were not confirmed, these exhibited biallelic transcription in MEFs, 14 of these are known to be tissue-specifically imprinted, 4 have very limited information available, and 1 gene, and *Dlk1* was paternally biased but was just below the cutoff with 21–25% leaky expression from the maternal allele.

### Imprinted transcript extensions

**Figure 2.**
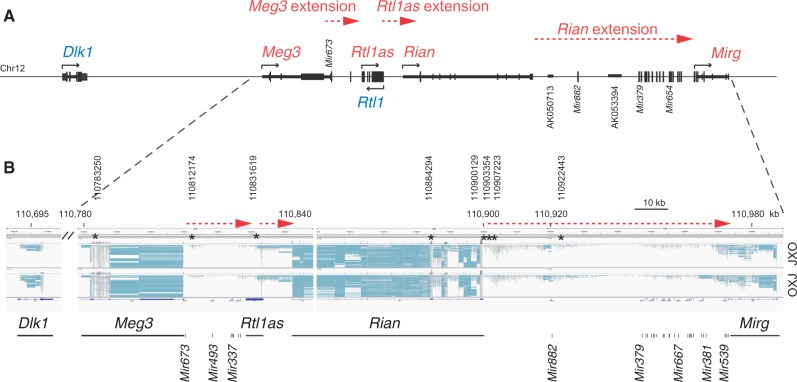
Extended transcription downstream of *Meg3*, *Rl1as* and *Rian.* **(A)** Map of the *Dlk1-Dio3* imprinted domain in chr12. Maternally (red) and paternally (blue) expressed transcripts are depicted with exon–intron structures and transcription orientation (arrows). **(B)** IGV screenshot of JXO and OXJ MEFs, as indicated to the right, is shown depicting the maternally expressed transcript extensions (red dotted arrows) downstream of *Meg3 (Gtl2)*, *Rtl1as* and *Rian*. Reads are shown in gray, splicing events are shown in horizontal blue lines. SNPs are colored vertical bars (visible only in high magnification). Asterisks indicate the SNPs used for sequenom allelotyping. Please refer to the online version for color information.

In addition to correctly predicting 32 known imprinted genes, we detected parental allele-specific transcripts that extended beyond known imprinted genes (Table 2; Supplementary Table S2). Each of these extensions was represented by a minimum of 2 and a maximum of 209 SNPs. A total of 267 SNPs predicted eight imprinted transcript extensions. Using the Integrative Genomics Viewer (IGV) (27) we could see the alternative SNPs for each of the maternally and paternally expressed extensions in the JXO and OXJ MEFs, respectively, similarly to the SNPs of confirmed imprinted genes. For each of these extensions, the parental origin of the alternative SNPs suggested that the extensions are expressed from the same parental allele as the transcript it extends. Examples are shown in Figure 2 and Supplementary Figure S1. The *Dlk1-Dio3* imprinted domain had three of the maternal allele-specifically transcribed extensions, extending in the sense strand from *Meg3*, *Rtl1as* and *Rian* (Figure 2). The intergenic transcripts exhibited much lower levels than the transcripts of *Meg3*, *Rtl1as*, *Rian* and *Mirg* genes. The intergenic transcripts appeared to read across all the way from the beginning of *Meg3* to the end of *Mirg*. However, no transcript was detectable in the intergenic regions of *Dlk1-Meg3* and *Mirg-Dio3* (not shown). The extension of the maternally expressed *Grb10* imprinted gene is displayed in Supplementary Figure S1A. The paternally expressed *Snrpn/Snurf* gene was extended toward the paternally expressed *D7Ertd715e* transcript (Supplementary Figure S1B). The paternally expressed *Nespas* RNA (28,29) extended beyond the *Mir296* and *Mir298* microRNAs (Supplementary Figure S1C). In addition, *Peg3* and *Peg12* paternally expressed imprinted genes were extended beyond their known 3′ ends (Table 2). The *Peg3* extended transcript is not annotated in RefSeq but corresponds to Ensemble gene prediction ENSMUST00000051209, encoding a 1571AA long protein. This predicted transcript is homologous with the human *PEG3* transcripts, which is 2 kb longer than the annotated mouse transcript.

**Table 2.** Imprinted transcript extensions detected in MEF deep sequencing

Transcript	Strand	Chromosome	Start	End	Number of SNPs	Average reads	Representative SNP	Reads JXO alt SNP	Reads JXO ref SNP	Reads OXJ ref SNP	Reads OXJ alt SNP	AF JXO	AF OXJ	FDR	Notes on IGV	Sequenom results
*Grb10* ext (N)	−	chr11	11824747	11830503	2	11	chr11_11828159	10	0	12	0	1.00	0.00	0.0182	MAT	
*Meg3* ext (N)	+	chr12	110809913	110828360	11	15	chr12_110826437	14	0	13	0	1.00	0.00	0.0006	MAT	MAT
*Nespas* ext (E)	−	chr2	174091931	174,106,739	15	23	chr2_174092928	0	24	0	22	0.00	1.00	0.0000	PAT	
*Peg12* ext (E)	−	chr7	69603714	69606757	2	16	chr7_69604676	0	10	1	23	0.04	1.00	0.0005	PAT	
*Peg3* ext (N)	−	chr7	6656607	6658671	7	68	chr7_6658505	0	52	0	69	0.00	1.00	0.0000	PAT	
*Rian* ext (E)	+	chr12	110899856	110967973	209	24	chr12_110903354	63	0	52	0	1.00	0.00	0.0000	MAT	MAT
*Rt1as* ext (N)	+	chr12	110831619	110842153	15	17	chr12_110841979	25	1	31	0	0.96	0.00	0.0000	MAT	
*Snurf* ext (N)	−	chr7	67119318	67126070	6	12	chr7_67121406	0	11	0	12	0.00	1.00	0.0044	PAT	

The imprinted transcript extensions detected in MEF deep sequencing are tabulated as in Table 1. Some of these imprinted extensions are novel (N), and others have been noticed before. We provide their full extent (E).

### Predicting novel imprinted genes

Of 1247 TRUE SNPs, only 123 predicted putative novel imprinted transcripts (Supplementary Table S2 and S4). Some predicted transcripts had no annotation in mm9 and we named them *MegDT1-MegDT5* and *PegDT1.* These transcripts presented a warning for such deep sequencing analysis. The mapping of these six transcripts with a total of 94 TRUE SNPs was ambiguous. *MegDT5* had an unusually high SNP coverage (∼30 SNPs along 1.1 kb) in chr14. Most of the SNPs occurred in both JXO and OXJ MEFs. The two TRUE SNPs, however, were exclusive to JXO MEFs, predicting maternal allele-specific transcription. We used NCBI blast to align the underlying sequence and found perfect match to the mRNA sequence of the paternally expressed imprinted gene, *Snrpn*. Because all the reads of *MegDT5* matched the *Snrpn* transcript but were different from the aligned sequence in chr14, we determined that we uncovered a silent pseudogene for the imprinted *Snrpn* gene on chr14. *MegDT1* with 66 SNPs was mapped to chr1 but had a paralogous region in the mitochondria chromosome (chrM). The remaining 28 TRUE SNPs predicted 22 unambiguously annotated novel imprinted transcripts with mostly 1 and maximum 3 SNPs each. When we looked at the annotated predicted transcripts in IGV, we found that even though the TRUE SNP indicated allele-specific expression, it was in minority among the SNPs along the same transcript. The majority of SNPs in the predicted transcripts appeared equally in both crosses, suggesting biallelic transcription. Therefore, these predicted transcripts were not genuine imprinted genes. For comparison we also display a few SNPs where the prediction program called a FALSE SNP even though the allelic frequency was at the 80% cutoff (Supplementary Table S2 and S4).

### Confirmation of the allele-specific expression using sequenom allelotyping

**Figure 3.**
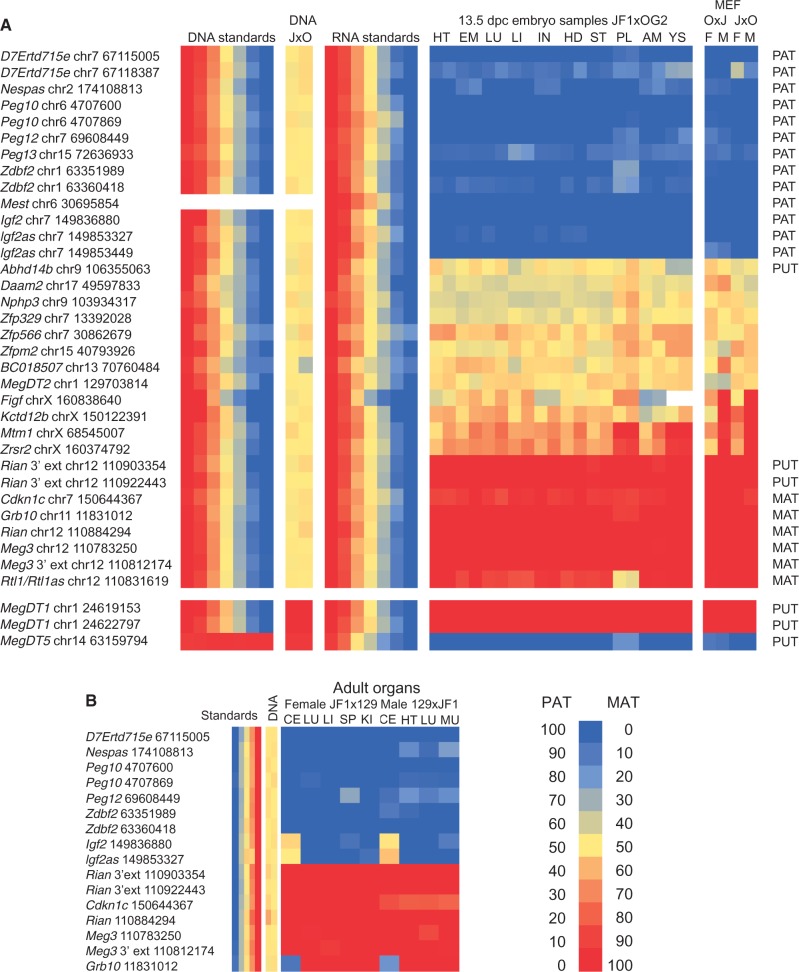
Analysis of parental-allele specific transcription by sequenom allelotyping. **(A)** Allele specific transcription was measured in the heart (HT), embryo carcass (EM), lung (LU), liver (LI), intestines (IN), head (HD), stomach (ST), placenta (PL), amnion (AM) and yolk sac (YS) samples in duplicates at 13.5 dpc and in female (F) and male (M) JXO and OXJ MEFs. After cDNA conversion, the ratios of JF1 and OG2 RNA alleles were measured using sequenom allelotyping at the SNPs listed to the left and are depicted in color from blue to red (0–100% maternal allele expressed in total expression). Standards and control are shown at the left panels of the heatmap. DNA standards: JF1 and OG2 DNA were mixed in the ratios of 1, 100:0; 2, 90:10; 3, 70:30; 4, 50:50; 5, 30:70; 6, 10:90; and 7, 0:100. The % of JF1 allele in the total (JF1 + OG2) alleles is calculated, and the levels are visualized ranging from blue to red (0–100%). DNA control: The JF1 and OG2 alleles are 50% each (yellow) in JXO female MEF DNA. RNA standards: JF1 and OG2 RNA from respective 13.5 dpc embryos were mixed in the ratios of 1, 100:0; 2, 90:10; 3, 70:30; 4, 50:50; 5, 30:70; 6, 10:90; and 7, 0:100. **(B)** Allele-specific expression was measured at the SNPs indicated to the left in adult JF1X129 female and 129XJF1 male organs cerebellum (CE), lung (LU), liver (LI), spleen (SP), kidney (KI), heart (HT) and muscle (MU). Standards: 129S1 and JF1 RNA from respective 13.5 dpc embryos were mixed in the ratios of 1, 100:0; 2, 90:10; 3, 70:30; 4, 50:50; 5, 30:70; 6, 10:90; and 7, 0:100. DNA control: 129XJF1 DNA was used for allelotyping.

We selected a subset of TRUE SNPs from the known and predicted putative imprinted genes for verification by multiplex sequenom allelotyping assays (Figure 3). We also included some controls from the FALSE SNPs. We used RNA from different organs of reciprocal JXO and OXJ 13.5 embryos and from JF1X129S1 and 129S1XJF1 adult organs to test the parental allele-specific expression.

Sequenom allelotyping confirmed that *Igf2* was paternally expressed in MEFs and 13.5 dpc organs (Figure 3A), but displayed biallelic transcripts in adult cerebellum (Figure 3B). This is expected based on the finding that *Igf2* is expressed from both parental alleles in adult choroid plexus and leptomeninges (30). Very little is known about the organ-specific expression pattern of *Igf2as* (31). Now we report that *Igf2as* transcription follows the same pattern as *Igf2*, being paternal allele-specific in MEFs as expected (23) and in each organ of the 13.5 dpc embryo (Figure 3A), but it is biallelic in the adult cerebellum (Figure 3B). This suggests that *Igf2* and *Igf2as* are under the control of the same parental allele- and tissue-specific regulatory elements. *Zdbf2* is a paternally expressed gene (32,33). Its transcript reads were ubiquitously paternal allele-specific in 13.5 embryos and adults, except that the placenta had a low level of transcripts from the maternal allele (Figure 3A). This latter may originate from contaminating maternal cells, which are not possible to remove completely from the placenta (15). Paternal allele-specific transcription of *D7Ertd715e*, *Nespas*, *Peg10, Peg12*, *Peg13* and *Mest* genes was confirmed in MEFs and in 13.5 dpc embryo and adult organs (except *Peg13* in the adult, because the *Peg13* SNP did not exist between 129S1 and JF1).

*Grb10* is an interesting imprinted gene affecting behavior, and exhibits a complex pattern of parental allele-specific expression, paternal allele-specific transcription in the fetal and adult brain and maternal allele-specific transcription in the placenta and other embryo organs (34,35). In MEF deep sequencing, *Grb10* transcript reads in the JXO cross displayed the alternative JF1 SNP variants, revealing maternal allele-specific transcripts (Figure 3A). Sequenom allelotyping confirmed *Grb10*’s maternal allele-specific transcription in MEFs and in each of the 13.5 dpc embryonic and other adult organs with the exception of the adult brain, where it exhibited paternal allele-specific expression (Figure 3A and B). The embryo head at 13.5 dpc displayed maternal allele-specific transcription, likely because the transcript level in the brain contributes to a small portion relative to the rest of the head (34). We confirmed the deep sequencing results for the maternally expressed *Cdkn1c*, *Meg3*, *Rian* and *Rtl1as* genes in the 13.5 dpc embryo and the adult. The SNP at *Rtl1*/*Rtl1as* detects two overlapping transcripts in opposite orientation. Maternal allele-specific combined transcription in each organ is mainly from *Rtl1as* and biallelic transcription in placenta is from maternal *Rtl1as* and paternal *Rtl1*, based on our previous observations using strand-specific analysis (36).

We confirmed the ubiquitous maternal allele-specific transcription of the *Meg3* and *Rian* extensions (Table 2; Figure 3). Sequenom allelotyping revealed, however, that the TRUE SNP at an unambiguously mapped putative imprinted gene, *Abhd14b* (Supplementary Table S4) was biallelically expressed in MEFs and also in 13.5 dpc embryo organs, confirming our observations in IGV. Similarly, biallelic expression was detected at the control FALSE SNPs (*Daam2*, *Nphp3*, *Zfp329*, *Zfp566*, *Zfpm2*, *BC018507*, *Figf*, *Kctd12b*, *Mtm1* and *Zrsr2*, which displayed an allelic frequency above the 80% cutoff in deep sequencing but failed the FDR cutoff (Supplementary Table S4; Figure 3). *Mtm1* and *Zrsr2* showed a very slight maternal allele-specific bias in embryonic organs, but were not imprinted in the classic sense. These two X-chromosome transcripts displayed correct paternal allele-specific inactivation in the placenta and yolk sac, while *Figf2* and *Kctd12b* did not. *MegD15* was mapped to chr1 but several chrM transcripts also aligned to this region. We tested the allele-specific expression of MegDT1 using allelotyping at two SNPs. We found ubiquitous maternal allele-specific expression (Figure 3A). However, only the maternal allele was represented in the genomic DNA in the heterozygous MEF lines. We determined that these transcripts were silent in chr1 but were expressed from maternally inherited mitochondria DNA. *MegDT5* mapped to the *Snrpn* pseudogene in chr14. In sequenom allelotyping, we found that this transcript was paternally expressed, suggesting that the pseudogene is silent and the reads belong to the paternally expressed *Snrpn* gene in chr7.

### Epigenetic analysis of the imprinted transcript extensions

The deep sequencing analysis of MEFs failed to detect novel imprinted transcripts apart from the transcript extensions in established imprinted domains. We wondered if these extensions are independent transcripts or they arise from missed termination of the known imprinted transcripts. We did not observe any deep sequencing reads between *Rian* and its extension, supporting the first possibility. However, RACE analysis at the junction of *Rian* and *Rian* extension successfully detected the 3′ end of Rian but failed to detect the 5′ end of the *Rian* extension (data not shown). In addition, reverse-transcription PCR amplified a fragment across the junction with an antisense primer coming from the extension. These results collectively suggested that the transcript between *Rian* and *Mirg* does not initiate at the 3′ end of *Rian* but is an extension of *Rian*.

**Figure 4.**
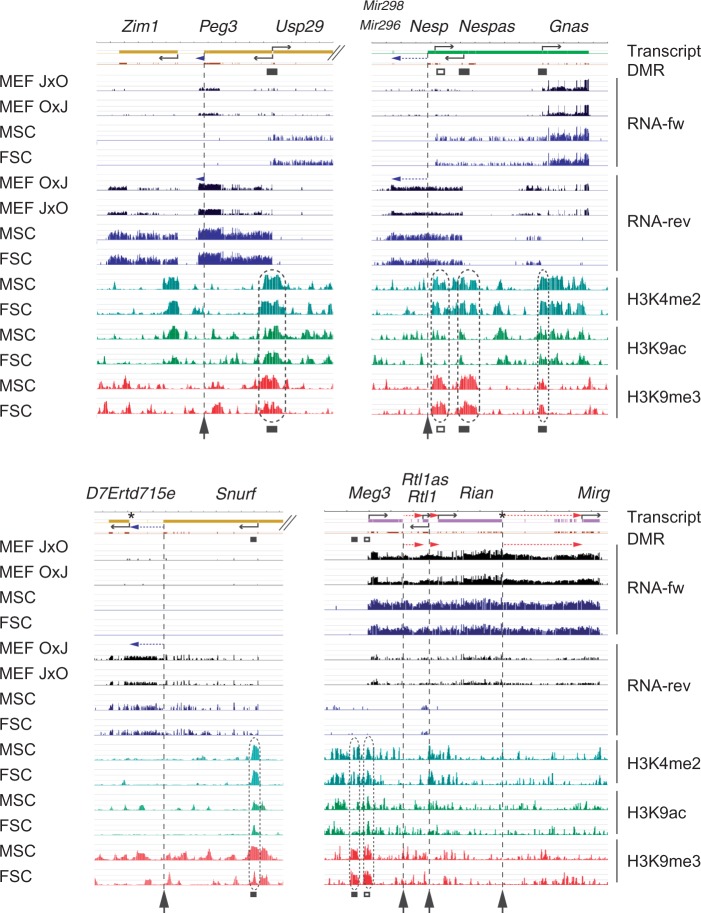
Chromatin analysis of imprinted transcript extensions. The location and orientation of known transcripts is shown at the top. The paternally and maternally transcribed imprinted transcript extensions are marked with blue and red horizontal dotted arrows, respectively. Germline- or somatic DMRs are labeled as black and open boxes, respectively. RNA deep sequencing results are displayed at four imprinted domains in forward (fw) and reverse (rev) directions in JXO and OXJ MEFs and female and male somatic cells (FSC and MSC) from fetal gonads at 15.5 dpc. The total reads were evenly scaled to 50 M and are depicted here in log_2_ scale ranging from 0 to 15. ChIP-chip results using the H3K4me2, H3K9ac and H3K9me3 antibodies are shown in FSC and MSC at 15.5 dpc. ChIP versus input DNA values are plotted as –log_10_ *P*-value scores in the range of 0–8.4. Note the opposite chromatin marking (dotted ovals) at DMRs and the lack of chromatin features at the 5′ end of imprinted transcript extensions (arrows pointing up along dotted vertical lines). Please refer to the online version for color information.

We wondered whether the imprinted transcript extensions are specific to MEFs or could be general components of known imprinted domains. To this end we compared the deep sequencing results obtained in JXO and OXJ MEFS with male and female somatic cells (MSC and FSC, respectively) of the embryonic testis and ovary at 15.5 dpc, as assessed recently (22). We found that each of the extensions also existed in MSC and FSC (Figure 4 and not shown), suggesting that they are not unique features of MEF. To get an insight of epigenetic regulation of the imprinted transcript extensions, we aligned the results of RNA deep-sequencing in MEF, MSC and FSC with ChIP-chip analysis (22). We found each of the known differentially methylated regions (DMRs) of imprinted domains marked by opposite chromatin marks in MSC and FSC (Figure 4). Specifically, we found the H3K4me2 and H3K9ac active marks together with the H3K9me3 repressive mark at each of the germline DMRs. This was expected based on previous studies that showed active and repressive chromatin marks in the DNA unmethylated and DNA methylated allele of the DMRs, respectively. However, the starting points of each extension displayed rather uneventful chromatin composition, suggesting that these are truly extensions of known imprinted genes and are under the epigenetic control of the DMRs of their respective domains.

**Figure 5.**
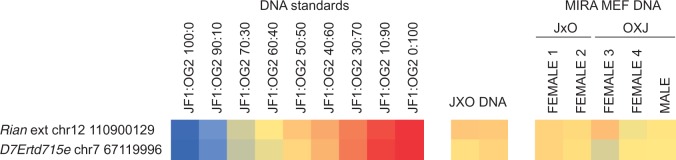
Analysis of parental allele-specific DNA methylation. MIRA-SNuPE analysis is shown in female and male OXJ and JXO MEFs as indicated at the 5′ end of the maternally expressed *Rian* extension and at the 5′ end of the paternally expressed *D7Ertd715e* gene. The methylated DNA fraction was collected, and it was subjected to allelotyping. The position of the SNPs used in allelotyping is marked in Figure 4 by asterisks. DNA standards: JF1 and OG2 DNA was mixed in the ratios indicated. JXO DNA: JF1X OG2 MEF DNA was subjected to allelotyping. Note that there is no evidence for parental allele-specific DNA methylation.

To detect parental allele-specific DNA methylation in the extended domains we performed Methylated CpG island recovery assay (MIRA)-SNuPE (20) at the promoter of *D7Ertd715e* and the junction of *Rian* and *Rian* extension, which could potentially serve as promoters. The methylated fraction was captured from the MEF DNA and was subjected to sequenom allelotyping at the specific genomic loci. We found no evidence for allele-specific DNA methylation at these positions (Figure 5) suggesting that the parental allele-specific transcription of *D7Ertd715e*, and *Rian* extension is secondary to the control of *Snrpn/Snurf* and *Rian*, respectively.

## DISCUSSION

MEFs are convenient sources for biochemical studies when cell number may be limiting from mouse embryos. To validate this model system with respect to imprinted gene expression, we carried out an allele- and strand-specific deep sequencing experiment using MEFs from reciprocal mouse crosses and performed validation using sequenom allelotyping of the same MEF samples, embryo organs from the same crosses and adult organs from two additional mouse crosses. We verified the parental allele-specific transcription of 32 ubiquitous imprinted transcripts. Based on the imprinting signature of MEFs, they provide valid models for understanding the biochemical aspects of genomic imprinting at these 32 imprinted genes.

Imprinted genes play important roles in development (1). Our RNA deep sequencing approach detected eight imprinted transcript extensions in known imprinted domains. A previous *in silico* analysis found that several intergenic ESTs in the *Dlk1-Dio3* domain transcribed in the same direction as *Rian.* They confirmed maternal allele-specific expression in one EST, CE2, downstream of *Rtl1as* (37). This intergenic transcript is identical to the *Rtl1as* extension we detected. Another study used selective priming and parallel sequencing in 9.5 dpc embryos and detected paternally expressed extension of *Nespas*, *Peg12* and maternally expressed extension of *Rian* and another maternally expressed transcript upstream of *Grb10* (38). Two additional fragments that showed maternal-allele specific transcription in uniparental mouse fibroblasts in the *Rian-Mirg* intergenic region (AK050713 and AK053394) (39) map to the *Rian* extension we observed in MEFs. Our deep sequencing discovered novel maternally expressed downstream extensions of *Grb10* and *Meg3*, and novel paternally expressed extensions of *Peg3* and *Snurf/Snrpn*. In addition, we were able to define the full extent of the eight imprinted extensions. It will be interesting to find out whether the imprinted transcript extensions play any function in development. Proper expression of imprinted genes along the *Dlk1-Dio3* imprinted domain is essential for development. Whereas paternally expressed coding transcripts are essential for perinatal viability, the maternally expressed non-coding RNAs play important role in postnatal viability (40,41). The level of pluripotency of induced pluripotent (iPS) cells correlated with the upregulated expression of this region (42). The role of the *Meg3* extension, *Rtl1as* extension and *Rian* extension will need to be tested in development and pluripotency using genetic experiments. The ubiquitously paternally expressed *D7Ertd715e* and the *Snurf* extension map to the *Snprn* imprinted domain, where loss of the paternal allele is implicated in the Prader-Willi syndrome. Again, further genetic studies need to test whether these imprinted transcripts, and the *Nespas*, *Peg3*, *Peg12* and *Grb10* extensions have a role in development or disease.

Small imprinted RNAs are recognized as regulators of biological functions (6). Imprinted transcript extensions in MEFs harbor miRNAs. Some of these miRNAs are imprinted, being expressed from the same parental allele as the extension. *Mir296* and *Mir298* are paternal allele-specifically expressed and their transcription in the paternal chromosome depends on the unmethylated allele of the germline DMR located at the *Nespas* promoter 27 kb away (8). Now we show that the paternally transcribed *Nespas* extension includes these miRNAs suggesting that it indeed serves as their precursor RNA. There is a large number of microRNAs along the *Dlk1-Dio3* imprinted domain, and all tested ones are transcribed from the maternal chromosome, including *Mir136* and *Mir127* along *Rtl1as, Mir370* along *Rian*, *Mir154, Mir337, Mir410* along *Mirg* and *Mir411, Mir380, Mir300, Mir376* and *Mir376b* along the *Rian* extension (9). Expression of all of these miRNAs requires the presence of the unmethylated allele of the IG-DMR in the maternal chromosome (9) similarly to the other maternally expressed non-coding RNA genes of the domain (43). Downregulation of the miRNAs of the *Dlk1-Dio3* imprinted domain coincides with downregulation of *Meg3* and *Rian* in iPS cells, and may contribute to their reduced pluripotency (44), as the efficiency of generating all-iPSC mice is diminished in case LOI of this locus occurs (45). It was speculated that the miRNAs are processed from a single precursor transcript initiating at the *Meg3* (*Gtl2*) promoter (9). Our deep sequencing and chromatin analyses support this hypothesis.

We revealed two potential caveats of using RNA deep sequencing method for discovering novel imprinted genes. The method has to be somewhat forgiving with respect to obtaining perfect alignments to detect SNPs. In certain cases allele-specific reads can detect silent pseudogenes of imprinted genes or may align to sequences of mitochondria origin that occur along autosomes. Our results stress the importance of careful observation of the data and thorough validation of imprinted expression using independent methodologies.

The JF1 versus OG2 comparison performed well for identifying imprinted transcripts. Even though the JF1 inbred line is less distant genetically from the reference genome than other non-domestic clad inbred lines, CAST/Ei or SPRET/Ei, its genome still contains 15 million SNPs over the C57BL/6J reference genome (25). The specific mouse crosses used in this study only prevented us from assessing the imprinting status of 4% (5 of 117) known imprinted genes due to lack of SNPs between OG2 and JF1. Fourteen of 19 imprinted genes that we did not confirm are known tissue-specifically imprinted genes. The other five that exhibited biallelic transcription in MEFs are likely tissue-specifically imprinted in cell types other than MEFs. In 61 cases, we were unable to make a call because of the insufficient transcript levels in MEFs. In summary, we can estimate by extrapolation, that using our method we should be able to detect at least 96% of imprinted genes if they were transcribed in a given cell type and if they exhibited imprinted transcription in that cell type. This method should be suitable for efficient screening of other mouse cell types or tissues.

Our RNA deep sequencing has confirmed 32 ubiquitous imprinted genes but identified no new bona fide imprinted genes in MEFs. In the light of our current results and other recent studies (13,15,23,46), we can safely predict that the final number of ubiquitously imprinted genes will not be more than 100. However, additional organs and cell types will need to be analyzed systematically to reveal the total number of imprinted genes exhibiting tissue or cell-specific pattern of imprinted expression.

## ACCESSION NUMBERS

MEF Deep sequencing data were deposited into GEO database with Series number: GSE49538.

## SUPPLEMENTARY DATA

Supplementary Data are available at NAR Online.

## FUNDING

National Institutes of Health (NIH) [RO1GM064378 to P.E.S.]; City of Hope [Excellence Award to P.E.S.]. Funding for open access charge: NIH [RO1GM064378 to P.E.S.].

*Conflict of interest statement.* None declared.
